# Experiences and Perceptions of Older Adults Participating in Physical Exercise, Cognitive Training, and Health Education Programs: A Qualitative Study Nested in a Randomized Controlled Trial

**DOI:** 10.3390/healthcare14193171

**Published:** 2026-09-24

**Authors:** Laura Pilar De Paz-Montón, Juan Manuel Carmona-Torres, Víctor Serrano-Fernández, Paulo Santos-Costa, Beatriz de Oliveira-Xavier, José Alberto Laredo-Aguilera

**Affiliations:** 1 Faculty of Physiotherapy and Nursing, University of Castilla-La Mancha, 45004 Toledo, Spain; laurapilar.paz@uclm.es (L.P.D.P.-M.);; 2 Multidisciplinary Research Group in Care (IMCU), University of Castilla-La Mancha, 45004 Toledo, Spain; 3 Institute of Health Research of Castilla-La Mancha (IDISCAM), 45004 Toledo, Spain; 4 Department of Nursing, Faculty of Medicine, University Autónoma of Madrid, 4 Arzobispo Morcillo Street, 28029 Madrid, Spain; 5 The Health Sciences Research Unit: Nursing (UICISA-E), Nursing School of Coimbra, University of Coimbra, 3004-531 Coimbra, Portugal

**Keywords:** older adults, sleep, healthy aging, physical activity, cognitive training

## Abstract

**Background:** Multimodal interventions combining physical exercise and cognitive stimulation may improve sleep and healthy aging among older adults. However, little is known about how participants experience these interventions. This study explored the experiences and perceptions of older adults who participated in physical exercise, combining physical exercise and cognitive training, or health education programs designed to improve sleep. **Methods:** A qualitative process evaluation using reflexive thematic analysis was conducted following a randomized controlled trial in Toledo, Spain. Twenty-one community-dwelling adults aged ≥65 years from the control, physical exercise (Otago), and combined exercise plus CogniFit^®^ groups completed semistructured interviews. The interviews were audio-recorded, transcribed from the recordings, and analyzed using reflexive thematic analysis. The study was reported in accordance with the Consolidated Criteria for Reporting Qualitative Research (COREQ) guidelines. **Results:** Six themes emerged: participation experience, perceived changes in sleep, benefits beyond sleep, evaluation of program components, facilitators and barriers, and maintenance of healthy habits. The participants reported high levels of satisfaction across all the groups. Beyond perceived changes in sleep, participants described perceived benefits related to physical well-being, cognitive stimulation, social interaction, and healthier habits. The participants also identified facilitators and barriers affecting engagement and maintenance. Exercise was valued for its simplicity and adaptability, whereas cognitive training was considered stimulating despite initial technological challenges. The main barriers were preexisting health conditions and difficulties using digital technology. Many participants intended to maintain the healthy habits acquired during the intervention. **Conclusions:** The participants who completed the qualitative interviews generally described positive experiences across the three groups. These findings provide insights relevant to the design of person-centered and accessible interventions and highlight factors that may influence engagement and maintenance in community settings.

## 1. Introduction

Sleep is a fundamental physiological state related to physical, cognitive, and emotional well-being. In older adults, sleep quality and quantity are often affected, as age leads to a decrease in REM sleep, an increase in night-time awakenings, increased daytime sleepiness, and a greater frequency of naps, creating a cycle of imbalances that perpetuate poor sleep quality [1]. In addition to age-related physiological changes, sleep problems in older adults are associated with increased frailty, functional decline, cognitive impairment, depressive symptoms, reduced quality of life, and an increased risk of falls and dependence [2,3]. Owing to their high prevalence and impact on healthy aging, sleep disorders constitute a significant public health problem in this population [4].

In older adults, the management of insomnia and other sleep disorders continues to rely frequently on pharmacological treatments, primarily benzodiazepines and other hypnotic agents [5]. However, evidence has shown that prolonged use can be associated with undesirable effects, including the development of tolerance and dependence, as well as increased daytime sleepiness and other geriatric syndromes [6]. Given these limitations, nonpharmacological interventions have become increasingly important as strategies aimed at improving sleep quality and promoting healthy aging [7,8].

Among nonpharmacological interventions, both physical exercise programs and cognitive interventions have been demonstrated to improve sleep in older adults [9,10,11,12,13,14,15,16,17]. In this context, the Otago program has shown positive effects on functional capacity and balance [9,10,11,12], whereas computerized cognitive interventions, such as CogniFit^®^, have demonstrated improvements in some sleep parameters, such as sleep latency and efficiency [13,14,15,16,17].

Although the effectiveness of these interventions is usually evaluated using clinical indicators and objective outcomes, these approaches do not always capture how older adults experience the intervention or how they perceive the processes underlying changes in sleep [18]. Physical exercise and cognitive stimulation may improve sleep through multiple pathways, including increased daytime activity, reduced psychological distress, increased cognitive engagement, and the establishment of healthier daily routines [9,10,11,12,13,14,15]. In addition to their potential effects on sleep, participation in these interventions may influence subjective aspects relevant to older adults, such as emotional well-being, self-esteem, perceived self-efficacy, the establishment of healthy routines, and the strengthening of social relationships, all of which are closely related to quality of life and healthy aging [19,20].

Qualitative research has shown that older adults value physical activity interventions when they are meaningful, enjoyable, adapted to their abilities, and provide opportunities for social interaction. Previous studies have also identified factors influencing participation, including perceived benefits, motivation, accessibility, and barriers such as health limitations or technological challenges [21,22,23]. However, these studies have focused mainly on participation in physical activity or health promotion programs in general rather than on multimodal interventions specifically designed to improve sleep through the combination of physical exercise and cognitive stimulation [21,22,23]. Consequently, little is known about how older adults experience these sleep-focused interventions, how they perceive their influence on sleep and daily life, and which components they consider most valuable or challenging. Understanding these experiences is essential for complementing quantitative findings and guiding the design of interventions that are not only effective but also acceptable, meaningful, and responsive to the needs and expectations of older adults.

In recent years, interest in multimodal interventions for older adults has increased because of the belief that combining physical exercise and cognitive training could generate synergistic effects on physical, cognitive, and emotional health. Several studies have indicated that these multidomain interventions can promote healthier aging and contribute to improving variables related to sleep quality, cognition, and quality of life [24,25]. Despite the growing evidence regarding their effectiveness, less attention has been given to understanding how these interventions are experienced by participants, which components are perceived as most valuable, and which factors facilitate or hinder engagement. Such information is essential for understanding not only whether an intervention works but also how and why it works in real-world settings.

A preliminary randomized controlled trial examined the effects of physical exercise and cognitive training in adults aged over 65 years in the province of Toledo, Spain. The combined intervention (Otago exercise plus CogniFit^®^) was associated with significant improvements in objective sleep parameters, including sleep efficiency (*p* = 0.027) and wakefulness after sleep onset (WASO) (*p* = 0.031), whereas the physical exercise group showed a significant improvement in quality of life (EuroQol, *p* = 0.043). Although these quantitative findings provided preliminary evidence of beneficial effects, they did not capture participants’ experiences of the intervention, their perceptions of its different components, or the contextual factors that may have influenced engagement and perceived benefits. Qualitative exploration can therefore complement these findings by providing insight into how participants experienced the intervention and which factors may contribute to its perceived value and feasibility in real-world settings.

Therefore, a qualitative process evaluation was undertaken to complement the trial findings. Guided by the Medical Research Council framework for process evaluations of complex interventions [18] and using reflexive thematic analysis [26,27], the study sought to generate contextual insights into the acceptability, perceived value, and feasibility of the intervention, with particular attention to factors that may inform the development and implementation of future community-based sleep-promoting interventions for older adults.

### Main Objective

To explore older adults’ experiences and perceptions of the intervention programs delivered within the randomized controlled trial, the aim of identifying factors that may inform the optimization and implementation of future community-based interventions to promote sleep and healthy aging should be explored.

## 2. Materials and Methods

### 2.1. Design

This study is a qualitative process evaluation conducted within an interpretive qualitative paradigm as a follow-up to a randomized controlled trial entitled “Efficacy of a Nurse-Developed Physical and Cognitive Program to Improve Sleep and Quality of Life in Older Adults: A Randomized Clinical Trial”, conducted in 2025 and registered at ClinicalTrials.gov (Identifier: NCT07251452). This trial is currently under review for publication. While the randomized controlled trial evaluated the effectiveness of physical exercise and cognitive stimulation interventions on sleep quality and quality of life using quantitative methods in older people who wanted to improve their quality of sleep, the present study aimed to explore participants’ experiences and perceptions of the intervention programs, identify perceived mechanisms of change, and examine barriers and facilitators to participation. In doing so, this process evaluation complements the quantitative findings by providing a deeper understanding of how the interventions were experienced, which components were perceived as most valuable, and which contextual factors may influence the implementation of similar community-based interventions in the future.

Data were collected through semistructured interviews and analyzed using reflexive thematic analysis, following the approach described by Braun and Clarke [26]. This approach was selected because it provides a flexible and theoretically informed method for developing patterns of shared meaning across participants’ accounts while recognizing the active and interpretive role of researchers in the construction of themes.

To ensure methodological quality and transparency in the conduct and reporting of the study, the Consolidated Criteria for Reporting Qualitative Research (COREQ) checklist was followed [28].

### 2.2. Subjects and Study Setting

Individuals over 65 years of age who had completed the dual program of physical activity and cognitive intervention in the province of Toledo entitled “Efficacy of a nurse-developed physical and cognitive program to improve sleep and quality of life in older adults: randomized clinical trial.”

The aforementioned program or study consisted of 3 groups:

- Control group: Underwent preintervention, postintervention, and 2-month follow-up assessments. They received 3 talks on health topics unrelated to sleep.
- Intervention Group 1: Participants completed the assessments and, for 12 weeks, performed the Otago physical training program exercises three times a week for 30–45 min.
- Intervention Group 2: Participants completed the assessments, the Otago physical training program, and cognitive training using the CogniFit^®^ smartphone app every 48 h for 12 weeks.

### 2.3. Inclusion and Exclusion Criteria

Participants were eligible if they had provided written informed consent and had sufficient exposure to the intervention to be able to reflect on their experiences. This was defined as attendance at least two of the three educational sessions in the control group and completion of at least 80% of the physical exercise sessions (intervention group 1) or the combined physical exercise and CogniFit^®^ sessions (intervention group 2). This eligibility criterion meant that participants who discontinued the program or had insufficient exposure were not eligible for the qualitative interviews. Participants with moderate-to-severe cognitive impairment evaluated by the Mini-Mental State Examination or who were unable to communicate fluently in Spanish were excluded.

### 2.4. Sampling

A nonprobability convenience sampling strategy was used, and adults aged 65 years and older who had previously participated in the randomized controlled trial evaluating the effects of physical exercise and cognitive training on sleep were recruited. Eligible participants who voluntarily agreed to participate and provided written informed consent were included. Participants were recruited from all three trial arms (control, physical exercise, and combined physical exercise + cognitive training) to capture a range of experiences across the intervention programs.

Sample adequacy was guided primarily by the concept of information power. The adequacy of the sample was considered in relation to the focused aim of the study, the specificity of the participant sample, the use of focused semistructured interviews, and the planned reflexive thematic analysis. Recruitment was therefore continued until the research team considered that the dataset provided sufficient experiential depth and contextual information to address the study aims across the three trial groups. No criterion of data saturation based on the emergence of new codes or themes was used to determine the final sample size.

### 2.5. Recruitment

Recruitment was conducted by telephone between 1 and 3 months after the program was completed. Participants were informed of the reason for the call and the objective of the qualitative study to determine their ability to participate. Potential participants subsequently received information about the study, including the research objectives, the voluntary nature of participation, data confidentiality, and data processing. Only those participants who provided informed consent were interviewed.

### 2.6. Data Collection Technique

Data were collected through semistructured individual interviews (Supplementary Material) with older adults of both sexes who had participated in the randomized controlled trial. Each interview lasted approximately 10–15 min. The semistructured interview methodology was selected because it allowed for a specific approach to the topic of sleep and quality of life while maintaining the necessary flexibility for the researcher to delve deeper into relevant aspects that arose during the conversation and explore the participants’ experiences and perceptions in greater detail [29].

Although the interviews were relatively brief, the interview guide was designed specifically to address the main dimensions of the intervention and participants’ experiences. The focused nature of the interviews, together with the use of open-ended questions and follow-up prompts when relevant, allowed participants to provide information directly related to the study objectives. The dataset was considered sufficiently rich to support the development of the six themes and their related subthemes and to address the research questions across the three trial groups.

For the control group participants, the interviews included questions aimed at understanding their feelings and perceptions about the assessments carried out and the educational talks received, as well as the possible influence of these activities on their night-time rest. Their opinion on the possibility of having participated in a physical exercise and cognitive training program aimed at improving sleep was also explored.

The interviews were conducted by de Paz-Montón, a researcher who had received prior training in qualitative research methods. The interviewer had previously delivered the intervention as part of the randomized controlled trial; therefore, a preexisting relationship with the participants had been established before the qualitative phase. This prior relationship may have influenced the data collection process, as participants may have felt inclined to provide socially desirable responses, particularly positive evaluations of the intervention, or may have been reluctant to express criticism. To minimize this potential influence, participants were explicitly informed at the beginning of each interview that the purpose of the study was to understand their experiences openly and honestly, that both positive and negative opinions were equally valuable, and that their responses would be treated confidentially and would not affect their relationship with the research team or any future participation. The interviewer also adopted a nonjudgmental and open approach throughout the interviews, allowing participants to express both favorable and unfavorable experiences freely.

### 2.7. Procedure

The interviews were conducted individually at senior centers in the province of Toledo where the previous study was carried out. A quiet and private environment was chosen to ensure the participants’ comfort and the confidentiality of the information; only the interviewer and the participants were present. Informed consent was obtained from all participants prior to the interview. The interviews were not returned to the participants for feedback or corrections.

The interviews were audio-recorded with the participants’ prior authorization.

### 2.8. Data Analysis

#### 2.8.1. Transcription

The interviews were audio-recorded using Microsoft Teams with the participants’ prior authorization at senior centers in Toledo and transcribed from the recordings. The transcription preserved the substantive content and meaning of the participants’ accounts. Nonsubstantive filler words, repetitions, and expressions that did not contribute to the meaning of the account were omitted to improve readability. No substantive statements or content relevant to participants’ experiences and perceptions were intentionally excluded.

Each participant was identified using an alphanumeric code (P1, P2, P3…). Participants’ identifying information was anonymized.

A second researcher (JMCT) reviewed the transcripts to support familiarization with the dataset and to facilitate reflexive discussion during the subsequent analytical process. This review was not intended to verify transcription accuracy or establish a single correct version of the participants’ accounts.

#### 2.8.2. Coding and Thematic Development

The analysis followed the principles of reflexive thematic analysis described by Braun and Clarke [26]. The first author (LPPM) became familiar with the transcripts through repeated readings and generated initial codes inductively, focusing on participants’ accounts of their experiences, perceptions, and factors influencing participation. Codes were subsequently developed and organized into broader patterns of shared meaning, which were progressively interpreted and developed into candidate themes and subthemes.

The analysis was iterative rather than linear, with movement between the transcripts, codes, candidate themes, and the developing interpretation. Negative, neutral, and divergent accounts were actively considered throughout the analysis, including accounts in which participants reported no perceived benefit or attributed changes to factors other than the intervention. These contrasting accounts contributed to the refinement and contextualization of the developing themes.

Participants’ accounts were also considered across the three trial groups to explore similarities and differences in their experiences. Experiences shared across groups were distinguished from experiences specifically related to physical exercise, cognitive training, or health education components. Data were managed using ATLAS.ti v9.

#### 2.8.3. Reflexive Analytical Discussions

The initial coding was performed by the first author (LPPM), who was directly involved in the data collection and had previously delivered the intervention. A second researcher (JMCT), experienced in qualitative research, subsequently engaged with the development of the analysis through discussions of selected transcripts, codes, candidate themes, and interpretations. These discussions were not intended to establish coding reliability or agreement or to identify a single correct coding framework. Instead, they provided opportunities for reflexive engagement, questioning assumptions, considering alternative interpretations of participants’ accounts, and examining how the researchers’ perspectives and positions could shape the analytical process. The discussions contributed to the development and refinement of the final interpretation of the dataset.

#### 2.8.4. Methodological Rigor

Methodological rigor was supported through reflexivity, transparency in the analytical process, and detailed reporting of the study context and procedures. Reflexivity was maintained throughout the research process by considering how the researchers’ experiences, assumptions, and relationships with participants could shape data collection and interpretation. In particular, the first author’s previous involvement in delivering the intervention was explicitly considered as part of the interpretive context.

The research team engaged in reflexive discussions throughout the development of the analysis to question assumptions, explore alternative interpretations, and consider how different perspectives could shape the developing interpretation. Detailed descriptions of the study context, participant characteristics, recruitment strategy, data collection procedures, and analytical process are provided to help readers assess the relevance and potential transferability of the findings.

All the interviews were conducted, transcribed, and initially analyzed in Spanish. The participant quotations included in this manuscript were translated into English by the first author, who is fluent in both Spanish and English, and the translations were reviewed by a second bilingual researcher to assess their conceptual equivalence with the original transcripts. Back-translation was not performed, as the aim was to preserve the intended meaning rather than achieve literal equivalence.

### 2.9. Ethical Aspects of the Research

This study was approved by the Social Research Ethics Committee (CEIS) of the University of Castilla-La Mancha, number CEIS-2026-189419, on 16 April 2026. Furthermore, participants received information and an explanation of the study’s objectives and procedures, as well as a section allowing them to withdraw their participation at any time. The project adhered to good clinical practice guidelines, and all participants received information and provided their informed consent.

The research followed the fundamental principles of the Declaration of Helsinki, the Council of Europe’s Declaration on Human Rights and Biomedicine, and the UNESCO Universal Declaration. All participant data were treated confidentially in accordance with Organic Law 3/2018, of 5 December, on the Protection of Personal Data and Guarantees of Digital Rights, maintaining strict confidentiality and preventing access by unauthorized third parties. Members of the research team signed a confidentiality agreement.

## 3. Results

### 3.1. Participant Characteristics

A total of 22 participants were initially recruited for the qualitative component of the study. One participant did not attend the scheduled interview for personal reasons and therefore did not contribute qualitative interview data. The remaining 21 participants completed an interview, and all 21 interviews were included in the qualitative analysis. Among these participants, 8 were men and 13 were women (Table 1). Thus, the final analytical sample included 21 participants.

### 3.2. Overview of the Analysis

The analysis of the interviews identified six main themes that describe participants’ experiences during the program and their perceptions of the intervention’s impact on sleep, well-being, and daily life. Among the interviewed participants, accounts generally reflected a high degree of commitment to the intervention; however, the analysis also revealed divergent and negative experiences, including the absence of perceived changes in sleep, physical limitations, difficulties with some cognitive tasks, frustration related to the progressive complexity of the application, and challenges associated with maintaining healthy habits after the intervention. These contrasting accounts were considered alongside the positive experiences to provide a balanced interpretation of participants’ perspectives. Similarities and differences across the three trial groups were considered when the themes were interpreted. Experiences related to general aspects of participation, adherence, and perceived changes were compared across groups, whereas experiences specific to the physical exercise and cognitive training components were considered in relation to the groups receiving these interventions. The themes and subthemes are presented in Table 2.

The analysis considered the accounts of all 21 participants across the three trial arms. The number of quotations presented from each participant was not intended to be evenly distributed, as quotations were selected on the basis of their relevance, clarity, and ability to illustrate the meaning of the identified themes and subthemes. Some participants, particularly those in Intervention Group 2 (IG2), provided more detailed or illustrative accounts on specific aspects of the intervention and were therefore quoted more frequently. This greater representation in the quotations does not indicate that their views were given greater analytical weight. Rather, the themes and subthemes were developed through consideration of the dataset as a whole, with attention to convergent and divergent perspectives across participants and trial groups.

### 3.3. Participation Experience

#### 3.3.1. Motivation for Participation

The participants described various reasons for agreeing to participate in the study. Regardless of the group to which they belonged, the main motivations were related to curiosity, a desire to collaborate with the research, an interest in maintaining or improving their health, and the opportunity to acquire new knowledge about aging and sleep.

Participants in Intervention Group 1 (IG1) and IG2 stated that they saw their participation as a way to contribute to scientific advancement: “I found it interesting to participate... I wanted to contribute what I could to improve the study.” (P6, IG1). “The most beneficial thing is knowing that I was participating in a study... being a study subject seems interesting to me.” (P8, IG2).

On the other hand, another participant from IG1 stated that he was interested in knowing his state of health: “Because I like to look at my body and see how I’m doing. I’m getting older now, and I’m gradually losing muscle.” (P5, IG1).

In the control group, motivation was frequently related to an interest in learning more about certain diseases or problems associated with aging. Some participants explained that personal or family experiences with chronic illnesses sparked their interest in attending educational sessions.

One participant reflected on this: “My husband had a stroke, and it was also a way for me to learn slightly more.” (P16, CG).

Others from the control group (CG) indicated that they were motivated by learning how their abilities evolved with age: “It motivated me because it is a way for people like me to monitor our health and understand how we’re progressing at this age.” (P21, CG).

Overall, participation was perceived not only as contributing to a research study but also as an opportunity to learn about health and remain involved in activities beneficial to their well-being.

#### 3.3.2. Satisfaction with the Program

Most interviewees highly valued their participation experience, regardless of their assigned group. Expressions such as “I liked it,” “it was very interesting,” “it was worthwhile” and “I would participate again” appeared repeatedly throughout the interviews.

Participants from the IG1 and CG especially highlighted the treatment they received from the research team, the program’s excellent organization, and the feeling that the activities were adapted to the needs of older adults.

One participant commented, “I liked participating because I thought it was a good thing for people of a certain age.” (P20, CG).

Another participant stated, “I left happy; I found it easy and interesting.” (P18, CG). In addition, yet another said, “I liked participating. Whether I contributed more or less did not matter to me. I just wanted to give what I could to help others improve, and that is all.” (P3, IG1).

One particularly relevant aspect was that satisfaction with the program did not depend solely on experiencing improvements in sleep. Even those participants who did not perceive changes in sleep quality valued their participation positively for the opportunity to learn, stay active, or contribute to the development of research.

#### 3.3.3. Commitment and Adherence

In general, the participants demonstrated a high degree of commitment to the intervention. Most reported regular attendance at sessions, completion of the assigned activities, and a responsible attitude toward the study.

Several participants from IG2 indicated that both performing the exercises and using the assessment devices or cognitive activities became part of their regular routine during the intervention period.

One participant from group IG2 explained how their perception of cognitive training changed as the program progressed: “At first, they were difficult... then I got the hang of it, and in the end, I enjoyed doing them.” (P9, IG2).

Similarly, another participant indicated that using the sleep-tracking device caused no discomfort during the intervention: “The watch did not bother me at night; it was like I was not wearing anything. At first, I was hesitant to wear it since others usually bother me, but this one did not.” (P8, IG2).

Beyond regular attendance, participants’ accounts reflected a sense of responsibility toward the study, which appeared to reinforce their engagement throughout the intervention.

### 3.4. Perceived Changes After the Intervention

#### 3.4.1. Perceived Changes in Sleep

Although sleep was a central objective of the program, participants described perceived changes in sleep in relation to broader aspects of their physical and general well-being. The nature of these changes varied across groups and was not always attributed directly to the intervention.

In IG1 and IG2, participants generally described sleep as being interconnected with physical activity, daytime activity, healthy habits, and personal health circumstances. Several participants perceived improvements in sleep in the context of broader lifestyle changes rather than attributing them solely to the intervention.

“All of this also plays a part, even with this program. They’re complementary; one grain does not make a granary, but it helps the other. A little bit here and a little bit there, and it all adds up.” (P9, IG2). “I have lost some weight, I’m exercising a bit more, I have cut down on wine and beer… all of that has had an impact, and I have felt better.” (P9, IG2).

These accounts suggest that participants understood changes in sleep as part of a broader process of lifestyle modification rather than as the result of a single intervention component.

This broader perception of improved physical well-being was also reflected by a participant in IG1, who linked feeling better to the exercises and their perceived effect on maintaining agility, “Yes… I felt better.” (P3, IG1). When asked how she had improved, she attributed this feeling to the exercises and stated that they had allowed her to stay more agile and feel better during the intervention period.

One of the most frequent findings in both intervention groups was that many participants reported sleeping well before the study began. Consequently, they felt that there was little room for further improvement.

One participant in group IG1 clearly expressed this idea: “No, because I sleep well.” (P2, IG1).

Although she did not perceive significant changes in sleep quality, she did notice a slight modification in her sleep schedule: “Now I go to sleep at nine and I’m up by six or seven.” (P2, IG1).

Similarly, another participant in group IG1 explained that she had already incorporated relaxation routines before the study and that her sleep was satisfactory from the start: “The thing is, I have a system for falling asleep by relaxing. I do a relaxation exercise where I start with my brain, and then my whole body relaxes. That is what I did, and I fell asleep very well.” (P3, IG1).

These accounts illustrate how preexisting good sleep and established sleep routines could limit the perceived scope for improvement.

Participants also distinguished perceived changes in sleep from changes that they associated with illness, pain, or medical treatment.

A participant in group IG2 explained that during the study, she was undergoing a medical procedure that completely affected her sleep: “Yes, I have noticed changes, but not because of you, but because I am currently on a medical treatment that relieves my pain.” (P9, IG2).

In other cases, participants indicated that the program’s benefits were compatible with the influence of other illnesses or personal events. Thus, one participant in group IG2 acknowledged that the perceived improvement could not be attributed solely to the intervention: “Everything plays a part... the program too but also exercise, weight loss, and other changes I have made.” (P10, IG2).

Together, these accounts indicate that participants did not necessarily attribute changes in sleep to the intervention alone but considered them within a wider context of health and lifestyle factors.

#### 3.4.2. Physical Benefits

Participants from IG1 and IG2 described multiple changes related to their physical condition after the intervention. The most frequent improvements were related to mobility, flexibility, balance, and a reduction in musculoskeletal discomfort, aspects that facilitated the performance of daily activities.

Several participants noted that the exercises allowed them to feel more agile and maintain better physical fitness. One participant explained:

“I have noticed my body is slightly more flexible... I’m keeping my scoliosis under control with the exercises.” (P2, IG1).

Similarly, another participant indicated that the exercises had been especially beneficial in relieving the neck pain they experienced before starting the program:

“Doing these exercises, particularly the neck exercises, my pain went away within a week or two.” (P8, IG2).

These accounts indicate that perceived physical changes were related mainly to mobility, flexibility, and the relief of musculoskeletal discomfort.

#### 3.4.3. Cognitive Benefits

Participants in the combined group (IG2) described cognitive training through CogniFit^®^ as an activity that allowed them to keep their minds active through tasks requiring attention, memory, and concentration. Beyond the potential cognitive benefits, many interviewees positively valued the challenging nature of the application and the opportunity to exercise their minds in a way that differed from their usual activities.

One participant from IG2 noted that the application seemed interesting because “every so often there was a challenge” and acknowledged that when they failed to complete a task correctly, “it made me angry,” which increased their motivation to continue practicing (P8, IG2).

Other participants in IG2 highlighted that the application was entertaining and stimulating. One participant explained that they enjoyed doing these types of exercises because they were entertaining: “because it keeps me entertained. I mean, I prefer doing this to watching TV, the news, or series; this is more entertaining” (P9, IG2).

Similarly, some participants positively valued the opportunity to gain a better understanding of their own cognitive functioning through the proposed activities. One participant explained that they decided to participate because they wanted to “know how I was” to determine “what level I was at” and to test their cognitive performance through the exercises carried out during the intervention (P10, IG2).

Taken together, the participants’ accounts suggest that CogniFit^®^ was valued not only for cognitive stimulation but also for providing an engaging and personally challenging activity.

#### 3.4.4. Social Benefits

Beyond the physical and cognitive benefits (IG1 and IG2), the participants highlighted the social value of the intervention. Both the exercise sessions and the educational activities were described as spaces that fostered interaction with other people, the exchange of experiences, and a break from the daily routine.

One participant commented, “What I liked most is that it brought us together with people from here” (P2, IG1). This highlights the social dimension of participation beyond the intervention’s primary health objectives.

Furthermore, several interviewees stated that they preferred to perform the exercises in a group rather than individually. One participant explained that she would like to maintain these types of sessions “once or twice a week” because, by doing the activity with others, “it helps you concentrate” and encourages continued exercise (P1, IG1). The group format was also perceived as facilitating concentration and continued participation.

Other participants also noted that these types of initiatives helped foster contact among older adults. In this regard, one participant stated that activities such as this “improve people’s relationships” and felt that they should be organized more frequently (P6, IG1). Similarly, a participant from IG2 proposed expanding the range of programs aimed at older adults to facilitate greater social participation (P9, IG2).

In the control group (CG), the educational sessions were also positively valued as spaces to exchange experiences and resolve health-related questions with other participants.

Overall, the group format was perceived as an added value of the programs, providing opportunities for social interaction beyond their primary health-related objectives.

### 3.5. Evaluation of Program Components

In this section, the results are organized by intervention components (IG1: Otago physical exercise; IG2: Otago + CogniFit^®^; CG: educational session). The participants also provided specific evaluations of each program component. While the overall perception was positive across groups, the aspects most valued differed: the physical exercise program was described as simple and adaptable, the cognitive training as stimulating and challenging, and the educational sessions as useful and applicable.

#### 3.5.1. Evaluation of the Physical Exercise Program (IG1 and IG2)

Participants in groups IG1 and IG2 rated the Otago program very positively, highlighting primarily that the exercises were simple, safe, and adapted to their abilities. These characteristics facilitated their participation and contributed to their perception of the program as an accessible intervention for older adults with varying functional levels. One participant from Group 1 (IG1) expressed it this way: “The exercises were easy; you could do them at home without any problem.” (P1, IG1). “Anyone my age or even older can do them easily.” (P10, IG2).

In addition to its simplicity, several participants highlighted that the program instilled confidence while performing the exercises, an aspect especially valued by those with physical limitations or a fear of injury.

“I’ve noticed it; I’ve noticed, I don’t know; my body is slightly more flexible.” (P2, IG1).

Furthermore, it was observed that the program’s guided structure helped maintain consistency in physical exercise. “I feel like I need it, and I keep it in mind; I’ve even shared it with other people.” (P8, IG2).

#### 3.5.2. Evaluation of Cognitive Training (IG2: CogniFit^®^)

Participants in group IG2 described the cognitive training as a stimulating experience that allowed them to exercise their minds through activities different from their usual routines. Although some exercises were demanding, this level of challenge was perceived by many as a motivating element of the program.

One participant explained, “I found it interesting because every so often there was a challenge, and responding to and trying to complete these exercises sparked my interest.” (P8, IG2). “Although I may have been less successful, the most beneficial thing has been CogniFit^®^, because of exercising my mind, because of doing different things and being slightly more alert in that sense, opening my mind and sharpening my mind.” (P10, IG2).

However, some participants described an initial feeling of frustration when certain tasks proved particularly complex, although this perception did not affect their overall assessment of the training. “Well, sometimes, it’s true that you get a little frustrated because there aren’t any difficult things.” (P10, IG2).

“As for the app, it was a bit difficult to understand how it worked at first, but once you got used to it, it was enjoyable and truly made you think.” (P12, IG2).

Overall, CogniFit^®^ was valued as both a cognitive stimulation tool and a personally challenging activity, although some participants experienced initial difficulties and frustration.

#### 3.5.3. Evaluation of the Educational Sessions (CG)

Participants in the control group rated the educational sessions very positively, highlighting primarily the clarity of the explanations, the practical usefulness of the content, and the opportunity to resolve doubts related to common problems during aging.

They particularly valued the clarity and accessibility of the educational content, as reflected in their descriptions of the sessions as easy to understand and clearly explained: “They’re good because they’re very easy to understand and enjoyable, so I’m glad…” (P17, CG). “Look, I liked them because you explained them very clearly, and honestly, those of us who are a bit older are also a little interested in all those topics, for example.” (P7, CG).

In addition, some participants noted that the sessions helped them reflect on their own lifestyle habits or personal experiences: “Well, first, because you came to do something about stroke, and my husband had a stroke, and it was also a way to learn slightly more about it because they do not truly give you much information.” (P16, CG).

Beyond acquiring information, participants described the sessions as opportunities to reflect on personal and family experiences related to health.

### 3.6. Facilitators and Barriers to Participation

The analysis of the interviews allowed us to identify several factors that facilitated or hindered participation in the program. These factors were related to both the characteristics of the program and personal and contextual aspects that influenced adherence and the participation experience.

#### 3.6.1. Facilitators of Participation

One of the most significant facilitators was that participants perceived the physical exercises as being adapted to their functional abilities and easily integrated into their daily routines. This suitability allowed most participants to perform the activities with confidence and without perceiving them as excessively demanding.

A major facilitator was the perceived suitability of the physical exercises for participants’ abilities. The participants described them as easy and appropriate for their age and health conditions: “I found the exercises easy” (P3, IG1) and “The exercises were very suitable for our age and our ailments” (P2, IG1). This perception was also expressed by participants with musculoskeletal problems, who emphasized that the exercises were well suited to their abilities: “Yes, I think they worked well for me. Of all the exercises I’ve done, these were the best suited to my abilities.” (P4, IG1).

In the combined group (IG2), where CogniFit^®^ was also used, the simplicity of the physical program was also positively valued: “They’re gentle, not traumatic.” (P8, IG2). In IG2, cognitive stimulation was also perceived as a facilitator of engagement: “I think they benefit us a lot, especially cognitively. Keeping our minds active truly helps.” (P14, IG2).

Moreover, the participants in the control group positively valued the educational talks because they considered them clear, useful, and applicable to common health problems: “You explained them very clearly.” (P7, CG) or “They were even more helpful for learning how to prevent them.” (P7, CG).

#### 3.6.2. Barriers to Participation

Barriers to participation arose mainly from health-related limitations and, in IG2, from difficulties associated with the increasing complexity of some cognitive tasks.

One participant from IG1 explained that her clinical situation had changed during the study: “I’m currently undergoing medical treatment, and that also has an impact.” (P3, IG1).

Other participants described specific limitations that made some exercises difficult, especially those related to balance or musculoskeletal conditions: “What I found most difficult was balance.” (P4, IG1). Another participant highlighted the impact of physical discomfort on participation: “The only things that made it difficult for me to participate were the knee pain I experienced on some days.” (P13, IG2). Despite these difficulties, most participants reported adjusting the intensity of the exercises rather than discontinuing them.

With respect to the cognitive training and assessments, participants consistently identified the puzzle tasks as the most challenging component of CogniFit^®^. As one participant explained, “For example, the most difficult part of CogniFit^®^ for me was the puzzles. I don’t know if I’ve completed many of them.” (P11, IG2). Another participant described how the increasing complexity and time pressure occasionally generated frustration: “The puzzle games… as you progressed, there was less time and they started adding more and more pieces. That is when I’d often think, Come on, just let me finish it!” (P14, IG2). These comments suggest that the progressive increase in task difficulty could be demanding and, at times, discouraging for some participants.

Although some participants adapted as they became more familiar with the application, the accounts indicate that increasing task complexity and time pressure could nevertheless generate frustration. As one participant stated, “Well, look, even though I might have been less successful, the most beneficial thing has been CogniFit^®^ because of exercising my mind, because of doing different things and being slightly more alert in that sense, opening my mind and sharpening my mind, that is what I liked most.” (P10, IG2).

Overall, participants’ accounts indicate that health-related limitations and increasing cognitive task complexity were the main barriers described, although their impact varied across participants.

### 3.7. Maintenance of Habits and Recommendations for Future Interventions

After the intervention ended, several participants reported having incorporated some of the habits acquired during the program into their daily lives. In the intervention groups, several participants indicated that they continued to perform the exercises that had been most beneficial for maintaining mobility or relieving physical discomfort, whereas others expressed their intention to continue using cognitive stimulation tools, as they felt these helped them keep their minds active.

Several participants in the intervention groups described incorporating some activities into their routines. One participant reported continuing the exercises because of their perceived benefit: “I’ve incorporated them because they make me feel truly good.” (P1, IG1). Another described maintaining both physical exercise and a more regular bedtime routine after the intervention: “Since the program ended, I have continued doing some of the exercises at home because they make me feel better. I have also tried to keep a more regular bedtime routine, which is something I did not do before”. (P15, IG2).

In IG2, participants also expressed interest in continuing cognitive training, although practical considerations could influence this intention: “I liked them. In fact, I was going to look into downloading it.” (P10, IG2). “Yes, definitely, I found it useful. For example, I truly liked the cognitive exercises. In fact, I was thinking about downloading the app myself. The only downside is that it is a paid app, but I will see.” (P14, IG2).

Participants from IG1 also identified the need for continued structure and regular sessions as a potential facilitator of maintenance: “Once or twice a week would be great.” (P1, IG1). “It would be better if you came a couple of days a week.” (P2, IG1).

With respect to the control group, one participant said, “I think people should keep doing it and stick with the program because many people eventually become tired and say, ‘Well, I’m just going to stop.’ However, they should not, it is not something they should give up”. (P19, CG).

Overall, the participants expressed interest in continuing the activities and extending similar programs to those of other older adults. Their accounts highlighted the perceived value of the ongoing structure, regular opportunities for participation, and accessibility as factors that may influence the maintenance of health-related habits beyond the intervention period.

## 4. Discussion

The findings of this qualitative study show that older adults perceived their participation in the intervention program aimed at improving sleep positively. Although some participants described perceived improvements in nighttime rest, the experience transcended the initial objective of the intervention. They also described perceived benefits related to physical well-being, cognitive stimulation, social interaction, and the acquisition of healthy habits. The participants also identified several factors that facilitated their participation and noted some barriers, mainly related to preexisting health problems and initial difficulties using technological tools.

The present findings can be interpreted through the multidimensional sleep health framework proposed by Tighe et al. [30], which conceptualizes sleep health as the result of interactions among biological, psychological, behavioral, and social domains rather than as an isolated symptom. Consistent with this perspective, participants described sleep as being influenced by multiple aspects of their daily lives, including physical health, mental stimulation, lifestyle changes, and social participation. Importantly, some participants did not perceive changes in their sleep or attributed such changes to factors other than the intervention, such as medical treatment, pain, or changes in lifestyle. This suggests that participants did not necessarily interpret sleep changes as the direct result of a single intervention component but rather as part of a broader and multidimensional process [30,31].

These findings also highlight that the success of programs aimed at improving sleep in older adults should not be evaluated solely through clinical outcomes. Process evaluations provide complementary evidence by revealing how participants interpret perceived changes, which intervention components they consider meaningful, and the contextual factors that influence engagement and adherence. This complementary perspective highlights the importance of considering participants’ experiences and the perceived usefulness of intervention components when evaluating multidomain sleep-health programs for older adults [32,33].

Among the participants who participated in the qualitative interviews, experiences were generally positive across the three trial groups, although the analysis also revealed divergent and negative accounts. However, the reasons underlying this difference in satisfaction differed. While participants in all groups valued the opportunity to engage with the study and contribute to research that could benefit other older adults, those receiving the exercise and cognitive training interventions additionally described perceived improvements in physical functioning, cognitive stimulation and sleep.

These results highlight that participation in health promotion programs stems from motivations that extend beyond individual benefits, incorporating components related to altruism, social commitment, and a sense of purpose. In this respect, our findings align with previous qualitative research showing that older adults participate in exercise and health promotion programs driven by a combination of personal and social motives. Beyond seeking to improve their health, they value the opportunity to maintain an active role in society, acquire new knowledge, and contribute to the development of future interventions for other people of their age, which encourage both participation in and retention within these programs [12,34,35].

Furthermore, the accounts of participants with high adherence suggest that tailoring interventions to the functional capabilities of older adults, together with clear communication about the study objectives, perceived usefulness of the activities, and ongoing support, were experienced as important factors facilitating engagement [36].

In this regard, our findings highlight the importance of designing person-centered interventions, in which creating a climate of trust and active participation are considered fundamental elements for the success of programs aimed at promoting healthy aging. This highlights the value of considering multiple dimensions of health when interventions for older adults are designed and evaluated [37,38].

With respect to the physical component, participants described an improvement in their physical fitness and confidence in performing daily activities, which is consistent with the evidence from the Otago program, which is widely recognized for improving functional capacity and balance in older adults [39]. Cognitive training was perceived as a stimulating and motivating activity that promoted the maintenance of mental activity, although some participants experienced initial difficulties that diminished as they became familiar with the application, which coincided with the application’s objective of working on cognitive health [40]. Furthermore, the social component emerged as one of the most highly valued benefits, as group activities facilitated interaction with others, the exchange of experiences, and a break from daily routine, aspects that the literature has linked to greater adherence to health promotion programs and improved psychological well-being in older adults [41].

The participants identified several factors that influenced their experience during the intervention; among the main facilitators were the adaptation of the exercises to their functional abilities, the simplicity of the proposed activities, and the perceived usefulness of the program. These results are consistent with those of previous studies that indicate that interventions adapted to the needs and capabilities of older adults may facilitate adherence and satisfaction [42]. With respect to barriers, the main difficulties were related to preexisting health problems and the initial adaptation to cognitive training using digital tools. Although the interviewed participants generally remained engaged despite these difficulties, they highlighted the need to offer individualized support, especially during the first sessions, particularly when digital technologies that may require additional support and follow-up were introduced [43,44].

Therefore, it is important to consider that the implementation of interventions aimed at older adults depends not only on their clinical effectiveness but also on their accessibility, ease of use, and ability to adapt to the characteristics of the participants [45,46]. Therefore, incorporating older adults’ perspectives into the design, implementation, and evaluation of health promotion programs may help identify meaningful intervention features and address barriers to participation. Beyond describing participants’ generally positive experiences, this study provides new insights into why older adults engaged with the intervention and which program components they perceived as most valuable. The participants highlighted benefits extending beyond sleep, including physical, cognitive, behavioral, and social gains, while they also identified specific barriers that influenced participation. By capturing these experiences, this process evaluation complements the effectiveness findings of randomized controlled trials and contributes to a better understanding of the mechanisms that may facilitate engagement and adherence in multidomain interventions. These qualitative findings add contextual and experiential information to the outcomes without providing independent evidence of effective intervention. They may inform the design and implementation of future community-based interventions by identifying factors related to accessibility, acceptability, engagement, and perceived value.

### Limitations and Strengths

This study has several limitations. First, the findings are based on a relatively small sample, as is common in qualitative research, and therefore are intended to provide an in-depth understanding of participants’ experiences rather than statistical transferability. The sample also showed some imbalance in terms of sex and educational background. These differences may have influenced participants’ experiences, perceptions, and ways of expressing their views, particularly regarding their understanding of the intervention and their engagement with its different components. Therefore, the findings should be interpreted with consideration of the characteristics of the sample, and the perspectives identified may not fully reflect those of older adults with different sociodemographic or educational profiles. The interviews relied on participants’ subjective accounts, which may have been influenced by recall bias and social desirability. In addition, the interviews were conducted by the same researcher who had previously delivered the intervention during the randomized controlled trial. Although participants were explicitly encouraged to share both positive and negative experiences, were assured that all their opinions were equally valuable, and were informed that their responses would remain confidential and would not affect their relationships with the research team, some degree of social desirability bias cannot be excluded. The interviewer’s previous involvement in delivering the intervention may also have influenced questioning, coding, and interpretation, although measures were taken to minimize this potential influence. Selection bias should also be considered. The qualitative sample included participants who met the predefined exposure criteria and voluntarily agreed to participate in the interviews. Consequently, participants who discontinued the intervention, had lower adherence or declined qualitative participation were not included. The findings therefore primarily reflect the experiences of participants who remained sufficiently engaged with the trial to meet the interview eligibility criteria and cannot be interpreted as representing the experiences of participants with lower adherence or those who discontinued the intervention. This selection may also have contributed to the generally positive perceptions observed among the interviewed participants and limits the extent to which the findings can be used to characterize overall intervention acceptability or feasibility. Furthermore, because the study was conducted in a single Spanish province, the generalizability of the findings to other settings should be considered with caution.

Despite these limitations, this study has several strengths. As a qualitative process evaluation embedded within a randomized controlled trial, it provides valuable insights into participants’ experiences, perceived mechanisms of change, barriers to and facilitators of participation, and factors relevant to the implementation of community-based sleep-promoting interventions for older adults. The inclusion of participants from the three trial arms enabled the exploration of a range of perspectives. Methodological rigor was supported through adherence to the COREQ guidelines, explicit reflexive consideration of the researchers’ positions, transparent reporting of the analytical process, and the use of reflexive thematic analysis.

## 5. Conclusions

This study explored the experiences and perceptions of older adults who participated in physical exercise, combined physical exercise and cognitive training, or health education programs. Among the participants who completed the qualitative interviews, the experiences of the intervention were generally positive and meaningful, with perceived benefits extending beyond sleep quality to physical well-being, cognitive stimulation, social interaction, and the acquisition of healthy habits. Moreover, some participants reported no perceived changes in sleep or attributed changes to factors other than the intervention, such as medical treatment, pain, or lifestyle changes.

The participants described the adaptation of activities to their abilities as a factor that facilitated engagement, whereas initial difficulties with technology use and some health problems were described as barriers to participation. Importantly, participants’ accounts revealed that perceived changes were not necessarily understood as isolated or directly attributable to a single component but rather as part of a broader process involving physical activity, cognitive stimulation, daily routines, and other personal circumstances.

The main contribution of this qualitative process evaluation is that it provides an experiential perspective that complements the quantitative assessment of the trial, showing not only whether changes occurred but also how older adults understood and attributed those changes, what they valued in the intervention, and which factors shaped their participation. These insights may help identify practical considerations for refining and implementing future community-based interventions targeting sleep and healthy aging among older adults.

## Figures and Tables

**Table 1:** Characteristics of the 21 participants who completed the qualitative interviews and were included in the qualitative analysis.

Participant ID	Group	Sex	Age	Education	Marital Status
P1	IG1	Female	75	Secondary	Single
P2	IG1	Female	78	Primary	Married
P3	IG1	Female	73	Secondary	Married
P4	IG1	Male	82	University	Widowed
P5	IG1	Female	74	Primary	Married
P6	IG1	Male	72	Secondary	Married
P7	CG	Female	69	Primary	Married
P8	IG2	Male	69	Higher	Married
P9	IG2	Male	70	Higher	Married
P10	IG2	Female	65	Primary	Married
P11	IG2	Female	68	Higher	Married
P12	IG2	Male	65	Secondary	Married
P13	IG2	Female	66	Primary	Married
P14	IG2	Female	66	University	Married
P15	IG2	Male	70	Primary	Married
P16	CG	Female	78	Secondary	Married
P17	CG	Female	90	Higher	Divorced
P18	CG	Male	70	Higher	Divorced
P19	CG	Female	79	Primary	Widowed
P20	CG	Female	69	Primary	Divorced
P21	CG	Male	72	Secondary	Divorced

CG: control group (talks on health topics). IG1: intervention group 1 (physical activity). IG2: intervention group 2 (physical activity + cognitive training).

**Table 2:** Themes and subthemes.

Themes	Subthemes
Participation Experience	Motivation for Participation
	Satisfaction with the program
	Commitment and Adherence
Perceived Changes After Intervention	Sleep Benefits
	Physical Benefits
	Cognitive Benefits
	Social Benefits
Evaluation of the interventions	Evaluation of the Physical Exercise Program (IG1 & IG2)
	Evaluation of Cognitive Training (IG2)
	Evaluation of the educational sessions (CG)
Facilitators and Barriers to Participation	Facilitators of Participation
	Barriers to Participation
Maintenance of habits and recommendations for future interventions	

Own elaboration.

## Data Availability

The data generated and analyzed during this study are not publicly available because of privacy and confidentiality considerations, as the qualitative interviews may contain potentially identifiable personal information. However, the data may be made available from the corresponding author upon reasonable request from qualified researchers, subject to ethical and data protection requirements.

## Supplementary Materials

The following supporting information can be downloaded at https://www.mdpi.com/article/10.3390/healthcare14193171/s1. Ref. [47] is cited in the Supplementary Material file.
